# Influence of Environmental Factors on the Variability of Archaeal Communities in a Karst Wetland

**DOI:** 10.3389/fmicb.2021.675665

**Published:** 2021-09-03

**Authors:** Ying Chen, Kairui Qiu, Ziyuan Zhong, Tao Zhou

**Affiliations:** School of Biotechnology, Guilin Medical University, Guilin, China

**Keywords:** Bathyarchaeota, Euryarchaeota, MCG-11, ANME-2d, karst wetland

## Abstract

Archaea are ubiquitous and play an important role in elemental cycles in Earth’s biosphere; but little is known about their diversity, distribution, abundance, and impact in karst environments. The present study investigated the effect of environmental factors on the variability of archaeal communities in the sediment of the Huixian karst wetland, the largest karst wetland in South China. Sediment cores were obtained from four sampling sites with different water depths and macrophyte inhabitants in both the winter of 2016 and the summer of 2018. The community analysis was based on PacBio sequencing and quantitative PCR of the archaeal 16S rRNA gene. The results showed that Euryarchaeota (57.4%) and Bathyarchaeota (38.7%) were dominant in all the samples. Methanogenic Methanosarcinales (25.1%) and Methanomicrobiales (13.7%), and methanotrophic archaea ANME-2d (9.0%) were the dominant Euryarchaeota; MCG-11 (16.5%), MCG-6 (9.1%), and MCG-5b (5.5%) were the dominant Bathyarchaeota. The community composition remained stable between summer and winter, and the vertical distributions of the archaeal phyla conformed to two patterns among the four sampling sites. In the winter samples, the archaeal 16S rRNA gene abundance was approximately 1.0E+10 copies/g of wet sediment and the Shannon index was 7.3±5, which were significantly higher than in the summer samples and in other karst environments. A correlation analysis showed that the moisture content and pH were the factors that mostly affected the archaeal communities. The prevalence of nitrate in the summer may be a key factor causing a significant decrease in archaeal abundance and diversity. Two features specific to karst environments, calcium-richness and weak alkalescence of the water supplies, may benefit the prevalence of bathyarchaeotal subgroups MCG-11, MCG-5b, and MCG-6. These results suggest that in karst wetlands, most of the archaea belong to clades that have significant roles in carbon turnover; their composition remains stable, but their abundance and diversity vary significantly from season to season.

## Introduction

Karst is a topographical feature characterized by distinctive landforms and underground drainage systems. Karst systems comprise ~15–20% of the earth’s continental area (Ford and Williams, 2007). They occur worldwide and have distinct climatic, lithologic, and geological structures. Karst systems have important social-ecological values (Beltram, 2016). The surface-underground hydrographic networks of karst regions are natural water reservoirs, which is important for maintaining hydrological balance, such as for flood storage and water supplies for humans, animals and plants (Ford and Williams, 2007; Hartmann et al., 2014; Beltram, 2016). Additionally, these systems may have a considerable carbon sink effect, accounting for one-third of missing carbon sinks globally (Liu et al., 2008; Cao et al., 2018).

Prokaryotic microbes have important roles in global biochemistry cycles, affect water quality (Mermillod-Blondin et al., 2015; Malki et al., 2020) and the dynamics of karst processes (Lian et al., 2011). However, archaea, a significant portion of the prokaryotic community in the karst environment, have not been well studied, especially for spatial and temporal variation. To date, archaea have been detected in various karst environments, including thermal karst springs (Borsodi et al., 2018), karst aquifers (Lazar et al., 2017; Hershey et al., 2018), caves (Legatzki et al., 2011; Ortiz et al., 2014; McDonough et al., 2016; Reitschuler et al., 2016; Zhao et al., 2018), sinkholes (Nold et al., 2010; Davis and Garey, 2018), karst lakes (Fillol et al., 2015), and karst estuaries (Menning et al., 2018). These studies suggest that, because of the complexity and diversity of karst environments, the archaeal community abundance, function, and structure vary among different karst environments.

Euryarchaeota is a commonly found archaeal phylum in karst environments that contains physiologically distinct groups such as strictly anaerobic methanogens and methanotrophs. Methanogens are methane-producing archaea, which have an important role in removing fermentation products such as acetate and hydrogen (Evans et al., 2019). Anaerobic methanotrophic archaea (ANME) are capable of oxidizing methane in the absence of oxygen by using alternative electron acceptors such as sulfate or nitrate. ANME populations are classified into three distinct groups, ANME-1, −2, and−3, and they play significant roles in regulating the global methane cycle (Knittel and Boetius, 2009). Conventionally methanogenic and methanotrophic species fall into eight orders in the phylum Euryarchaeota, namely Methanomicrobiales, Methanosarcinales, Methanobacteriales, Methanococcales, Methanopyrales, Methanocellales, Methanomassiliicoccales, and “Candidatus Methanophagales.” The ANME-2 and ANME-3 clusters of the archaea belonging to the order Methanosarcinales, and “Candidatus Methanophagales” were proposed to designate ANME-1 (Adam et al., 2017).

Previous research in different karst areas showed that the dominant order of methanogens was distinct. In the karst Lake Vilar and Lake Cisó (Banyoles, Spain), two archaea classes, Methanobacteria and Methanomicrobia, were dominant in planktic and sediment samples (Fillol et al., 2015), which included the orders Methanobacteriales, Methanocellales, Methanomicrobiales, and Methanosarcinales. In a coastal karst sinkhole, Hospital Hole (Florida, United States), the predominant methanogenic order was Methanococcales (Davis and Garey, 2018). In wet soil in two karst caves, Gaden Cave and Cathedral Cave (Wellington, Australia), Methanosarcinales was the dominant methanogen (McDonough et al., 2016).

Members of the phylum Bathyarchaeota are globally distributed and conspicuously abundant in anoxic sediments, however, knowledge of the ecology of Bathyarchaeota in the karst environment is poor. Species of Bathyarchaeota have long been known as the Miscellaneous Crenarchaeota Group or Crenarchaeota. To date, the isolation of a pure bathyarchaeotal culture has not been reported, so metabolism in this phylum has not been well studied. Genomic and physiological evidence suggests that these organisms can anaerobically utilize organic substrates such as detrital proteins, polymeric carbohydrates, or fatty acids (Zhou et al., 2018). The phylum Bathyarchaeota was briefly divided into 25 phylogenetically distinct subgroups (Kubo et al., 2012; Fillol et al., 2016; Zhou et al., 2018). Recent research suggests the unique karst lake environment may support some specialized Bathyarchaeota subgroups (Fillol et al., 2015). Considering the complexity and diversity of the karst environment, studies of the Bathyarchaeota in karst environments will improve our understanding of the phylogenesis, taxonomy, and ecology of the phylum Bathyarchaeota.

Karst wetlands share common hydrologic features with other karst systems, including calcium-rich and weakly alkalescent water supplies (Beltram, 2016), which may provide optimal niches for some specialized archaeal groups. However, there is still limited information on the archaea in karst wetlands. A better understanding of spatial–temporal effects would provide more insight into the ecological function of archaea in karst wetlands. Some previous investigations of archaea in other wetlands have shown that the archaeal communities were influenced by distinct spatial–temporal factors. In a free water surface flow constructed wetland near Lake Erhai in southwest China, the population density of archaea was adversely affected by high temperature, and the nitrate and carbon/nitrogen ratios had significant roles in shaping the overall archaeal community structure (Li et al., 2017a); while in the Mai Po Nature Reserve, a coastal wetland located at the northwestern region of Hong Kong, the seasonal dynamics of the archaeal community abundance were minimal, and pH was the predominant factor that shaped the archaeal community (Zhou et al., 2017).

The Huixian karst wetland is considered as the largest karst wetland in South China (Cai et al., 2009; Chen et al., 2020; Pan et al., 2020) and has been listed as a national wetland park since 2012. This wetland has crucial ecological functions with respect to the Li River, including flood control, water purification, and biodiversity support (Qin et al., 2020). Because of its significant social-ecological value to nearby regions, many recent studies have reported on the hydrology (Pan et al., 2020), soil properties (Li et al., 2017b), contamination (Chen et al., 2020; Huang et al., 2020), and microalgal and bacterial communities (Yan et al., 2020) of the Huixian karst wetland. However, knowledge of the archaea in this environment remains minimal.

In the current work, we studied the spatial (*i.e.*, among sampling sites and sediment depths) and temporal (*i.e.*, winter 2016 vs. summer 2018) variations of the archaeal community in the sediment of the Huixian wetland. For the first time, we conducted comprehensive studies in summer and winter and studied sediment cores taken at different depths at various sample sites. We aimed to evaluate (1) the abundance of the archaea in these environments (2) the predominant archaeal group in each sediment sample, and (3) the environmental factors affecting the archaeal community.

## Materials and Methods

### Study Area and Sampling Procedures

The Huixian karst wetland is located in the southwest of Guilin City in the Guangxi Autonomous Region, at an altitude of 150–160m. The mean annual temperature for this area is approximately 16.5–20.5°C, with an average annual rainfall of 1890mm (Huang et al., 2020). The Huixian wetland is in a transition zone between a karst peak-cluster plain and a karst depression area, where the main water supply comes from the surrounding mountains through subterranean streams. Our previous research revealed rich organic carbon in the wetland sediment from 10 to 40cm layer, constituting 5–13% of the sediment (Chen et al., 2017).

**Figure 1:**
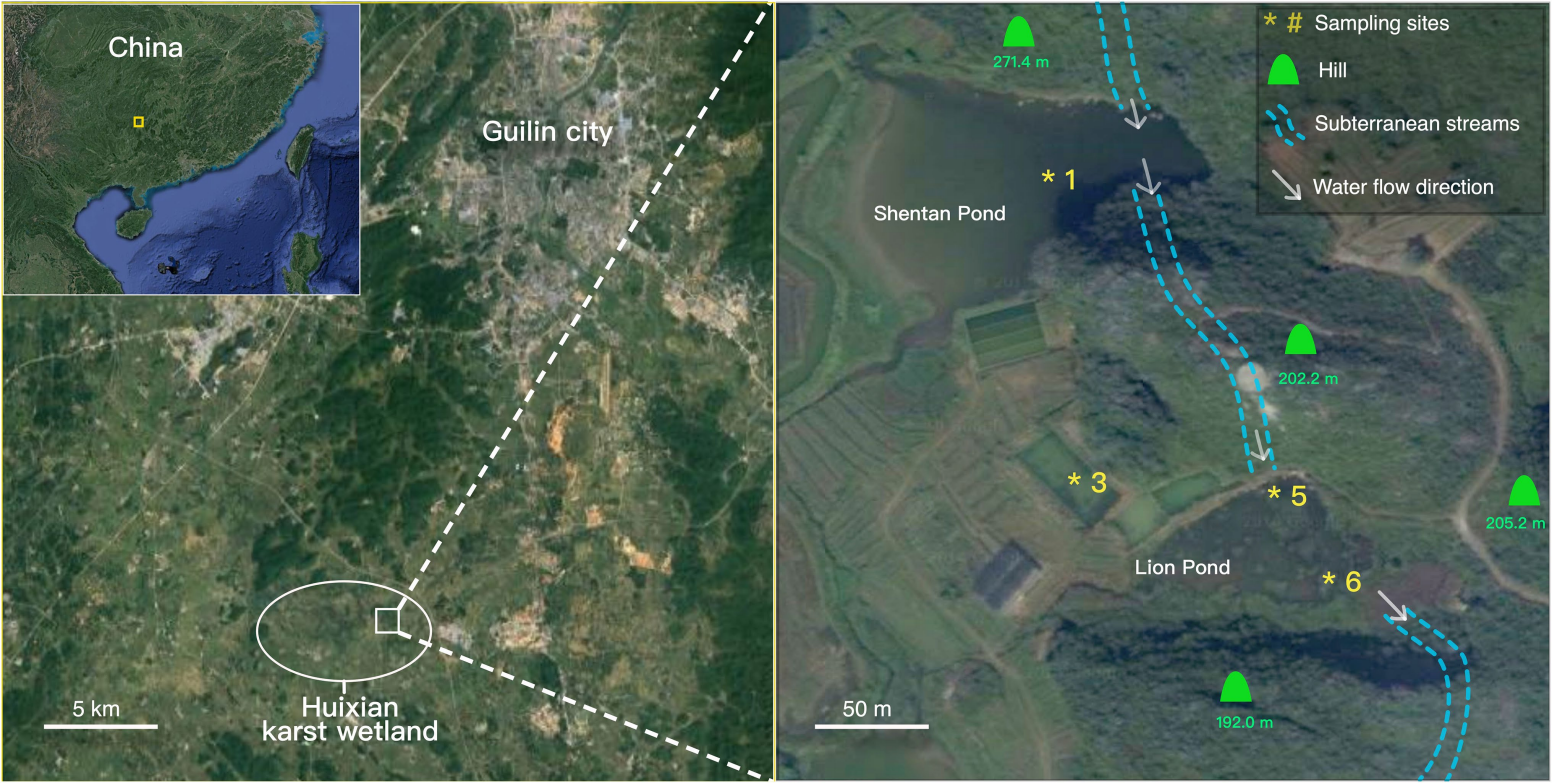
Satellite map depicting the Huixian Research Base for Karst Geology and the sampling sites based on Google satellite maps.

The sampling sites were within the Huixian Research Base for Karst Geology set up by the Chinese Academy of Geological Sciences. Sediment samples were collected using a cylindrical sediment sampler at four sites (sites 1, 3, 5, and 6; Figure 1) that had different water depths and macrophyte inhabitants. Winter samples were obtained in December 2016, and summer samples were collected in August 2018 at the same sites. Site 1 was located in Shentan Lake, approximately 9,364m^2^ in area, with an average depth of approximately 1.2m. Site 5 was in Lion Pond, whose water depth is approximately 0.5m. Site 3 was located at an artificial pond used for hydrophyte research; its water is channeled from Lion Pond, and its water depth was 0.7m. Site 6 was at the bay of Lion Pond located near the entrance of a subterranean stream that channels water out of Lion Pond. Both Shentan Lake and Lion Pond had a groundwater supply (dashed line in Figure 1). The water depth was the highest at site 1 (~1m), followed by sites 3, 5, and 6 at 0.7m, 0.5m, and 3cm (just covering the sediment), respectively. Submerged green plants inhabited the sediment of site 3 in the winter; these plants died in the summer because water hyacinths blocked the sunlight. No plants were observed at the other three sites.

After collection, each 30-cm-long sediment core was immediately divided into six pieces of 5cm each, transferred to sample cups, stored on ice, and delivered to the laboratory for DNA extraction and chemical analysis. Samples taken at site 1 were designated 1d1, 1d2, 1d3, 1d4, 1d5, and 1d6, respectively representing depth intervals 0–5, 5–10, 10–15, 15–20, 20–25, and 25–30cm from the top to the bottom layer of the sediment. The designations for summer samples started with “s” and for winter samples with “w.” For example, w1d1 represents 0–5-cm deep sediment from Site 1 collected in the winter. We collected 48 samples in total, and all samples were designated in this manner.

### Chemical Analysis

The pH of all sediment samples was determined using a HANNA HI99121 pH meter (HANNA instruments, Padova, Italy). The nitrate and sulfate concentrations in the sediment were measured by analyzing the pore water. To retrieve the diluted pore water for chemical analysis, 50g of sediment per sample was mixed with 70ml deionized water, and then filtered through 0.45-μm mixed cellulose ester membranes (Merck Millipore, Billerica, MA, United States). The nitrate, sulfate, and calcium assays were conducted by the China Nonferrous Metals Geology and Ming Limited Corporation (Guilin, China), following the Chinese standard HJ/T84-2016: “Determination of Water Inorganic Anions by Ion Chromatography.”

### DNA Extraction and PCR

The total genomic DNA was extracted from each prepared sediment sample using a Mag-Bind Soil DNA Kit (OMEGA Bio-Tek, United States). The DNA quality and concentration were analyzed *via* agarose gel electrophoresis and NanoDrop 2000 spectrophotometers (Thermo Fisher Scientific, Waltham, MA, United States).

Barcoded archaeal 16S rRNA gene amplicons were generated by a one-step PCR approach to decrease PCR bias. DNA samples were amplified directly using a set of 24 barcode-tagged archaea-specific 340F (CCCTAYGGGGYGCASCAG) and 1000R (GGCCATGCACYWCYTCTC) primers (Gantner et al., 2011). Supplementary Table S1 lists the barcode sequences. The amplicons were ~660bp long, covering the V3–V5 regions and a partial V6 region of the archaeal 16S RNA gene.

Every amplification reaction consisted of 10–50ng of DNA template, 25pmol of each barcode-tagged primer, 4μl dNTP mixture (200μM), 5μg bovine serum albumin (BSA), 1.5U ExTaq DNA polymerase, and 5μl 10×Ex Taq Buffer (Takara Bio Inc., Dalian, China). DNase-free ddH_2_O was added to a final volume of 50μl.

All PCR reactions were run in a Bio-Rad T100 thermal cycler (Bio-Rad Laboratories, Hercules, CA, United States) under the following conditions: preincubation at 95°C for 2min, followed by 10cycles of denaturation at 95°C for 30s, annealing at 51°C for 30s, elongation at 72°C for 45s and 25cycles of 95°C for 30s, 55°C for 30s, and 72°C for 45s, and a final extension step at 72°C for 7min.

PCR products were analyzed *via* agarose gel electrophoresis, and the fragments of the barcoded 16S amplicons were isolated from the gel using a MicroElute Gel Extraction Kit (OMEGA Bio-Tek, Norcross, GA, United States).

### DNA Sequencing and Data Processing

Two PacBio libraries were constructed; one contained a pool of barcoded 16S rRNA gene amplicons from 24 samples obtained in the summer, and the other included barcoded amplicons from 24 samples taken in the winter. The libraries were sequenced by Macrogen Inc. (Seoul, South Korea) on a PacBio RS system, using a Single Molecule, Real-Time (SMRT) cell 8Pac V3, DNA Polymerase Binding Kit P6 v2, following Template Preparation and Sequencing Protocol PN100-092-800-05. PacBio raw reads were processed by Macrogen Inc. using the RS_Reads of the Insert protocol in the SMRT Analysis software, version 2.3, to obtain demultiplexed consensus sequences with a minimum of three full passes. The Reads of the Insert data were deposited in GenBank as Sequence Read Archive PRJNA686692.

The Reads of the Insert data were subsequently processed using Geneious v10.2.6 (Biomatters Ltd., Auckland, New Zealand).^1^ Sequences of <600nt were discarded prior to downstream analyses. Chimeras were detected using UCHIME v4.2.4 with a reference dataset from Silva.^2^ Sequences were clustered into operational taxonomic units (OTUs) at 97% identity using the open-reference OTU-picking workflow of MacQIIME v1.9.1 (Cai et al., 2009), with SortMeRNA (Kopylova et al., 2012) and Sumaclust (Mercier et al., 2013) as the OTU-picking methods. Singletons were removed to prevent potential sequencing errors. Taxonomic assignment of each OTU representative sequence was performed using the Sequence Classifier tool in Geneious, from which representative sequences of each OTU were BLAST searched against a curated database specific to our dataset. For the taxonomic analysis of Bathyarchaeota, representative sequences of 25 previously reported Bathyarchaeota subgroups (Zhou et al., 2018) were added to the database.

### Quantification by qPCR

Copy numbers of archaeal 16S rRNA genes were determined from DNA samples by qPCR using the primers Arch806F/Arch958R (DeLong, 1992). Quantifications were run in a CFX96 cycler (Bio-Rad Laboratories). All reactions were performed in triplicate in a 25-μl reaction mixture containing 12.5μl of 2×SYBR Premix Ex Taq II (Takara Bio Inc., Dalian, China), 20–50ng template DNA, 200nM of each primer, 1μg BSA and DNase-free ddH_2_O added to a final volume of 25μl. The qPCR protocol was as follows: preincubation at 95°C for 2min, followed by 40cycles of denaturation at 95°C for 30s, annealing at 55°C for 30s, elongation at 72°C for 10s, and a final extension step at 72°C for 7min. After cycling amplification, the quality and length of the qPCR products were checked by a dissociation curve and 1.2% agarose electrophoresis. The plasmid pMD18-T-16S containing an insert of an environmental archaeal 16S rRNA gene isolated from the sediment of site 1 was used as the archaeal quantification standard. The qPCR results were expressed as the number of gene copies/g wet sediment sample (copies g^−1^ sample).

### Statistical and Phylogenetic Analyses

Alpha diversity indices, including OTUs, Chao1, ACE (abundance-based coverage estimator), PD (phylogenetic diversity) whole-tree indexes, Good’s coverage, Simpson’s index, and the Shannon diversity index, were calculated using MacQIIME, version 1.9.1 (Cai et al., 2009). A similarity-based analysis, ANOSIM (Clarke, 1993) and an analysis of indicator species (Dufrene and Legendre, 1997; Cáceres et al., 2010) were performed using the vegan and indicspecies packages in the R software. A Spearman correlation analysis, the Mann–Whitney test, and the Kruskal-Wallis one-way test were performed using GraphPad Prism v9.0.0 for Mac (GraphPad Software, LaJolla, CA, United States).^3^ A redundancy analysis and Bray-Curtis non-metric multidimensional scaling were performed using an online tool (Gene Denovo Co., Shenzhen, China).^4^ The 16S rRNA gene phylogenetic tree was reconstructed using the approximately-maximum-likelihood algorithm in FastTree 2.1.11 (Price et al., 2009).

## Results

### Spatial Profiles of Physicochemical Indicators

**Figure 2:**
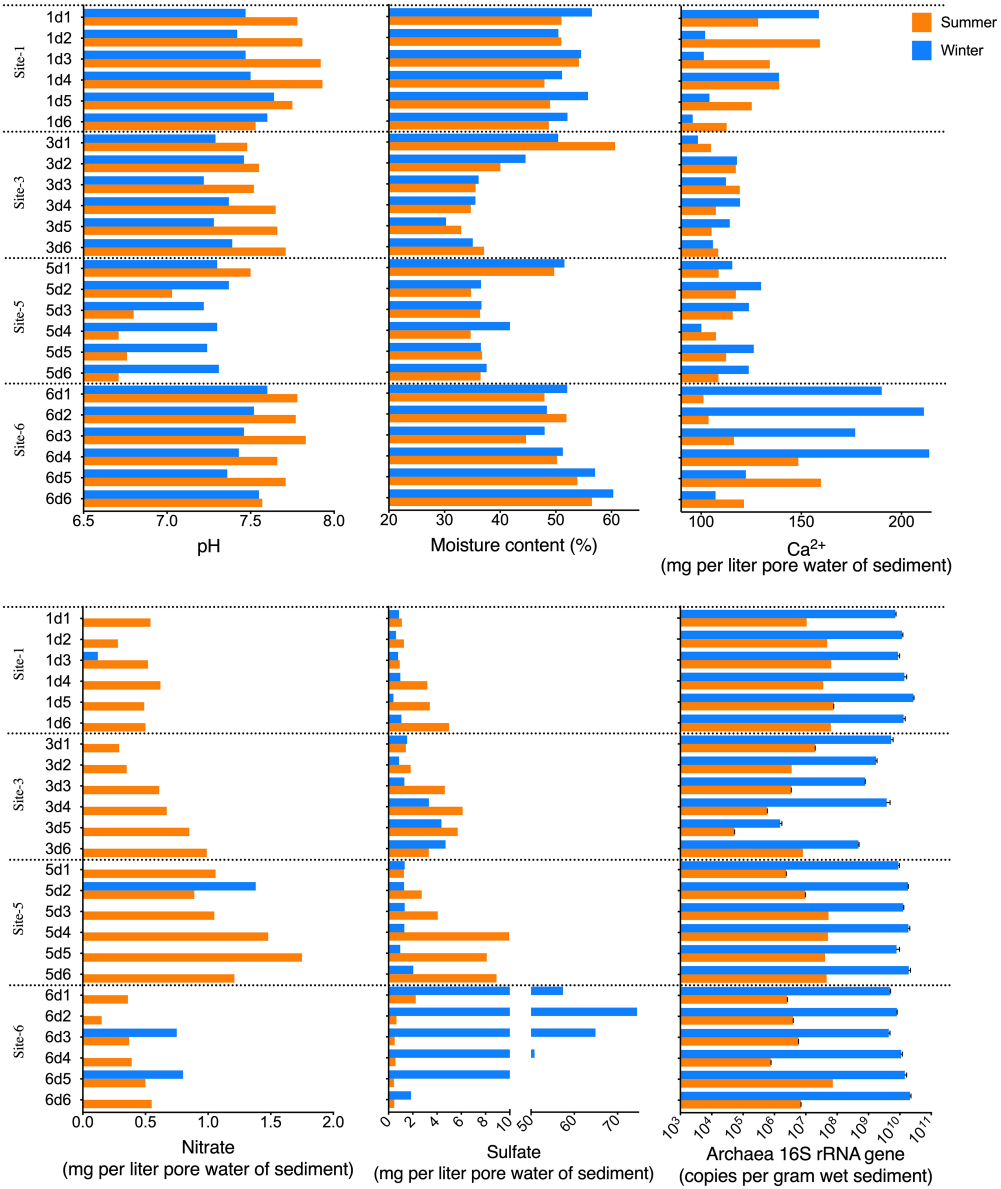
Physicochemical properties (pH, moisture content, and dissolved Ca^2+^, sulfate, and nitrate in the sediment pore water) and archaeal 16S rRNA gene abundance of the sediment samples.

The pH, moisture content, temperature, dissolved calcium ions, and nitrate and sulfate concentrations in each sample were analyzed as physicochemical indicators (Figure 2; Supplementary Table S2). Most of the samples were slightly alkaline (pH>7.0), and the dissolved Ca^2+^ in the sediment were approximately 87.38–213.83mg/l, agreeing with the typical water properties of a karst region.

Among the four sampling sites, sites 3 and 5 shared more common physicochemical profiles than they did with sites 1 and 6. Sites 1 and 6 showed a broader calcium concentration range than did sites 3 and 5. The vertical distribution patterns of the moisture content at sites 3 and 5 were similar, and the peak moisture content was found at surface sediment layer (0–5cm), and stayed at approximately 35% in the 5–30-cm sediment. However at sites 1 and 6, the moisture content did not change greatly from the 0 to 30-cm depth interval sediment, which was approximately 50%. The nitrate concentration at sites 3 and 5 showed an increasing trend with increasing sediment depth, and showed no clear trend at sites 1 or 6. Regardless of the vertical distribution profiles at site 1, the sulfate concentration in the samples from sites 3 and 5 also displayed an increasing trend from surface layers to the subsurface layers, while at site 6, a reverse trend with the depth profile was seen.

In addition to the data presented in Figure 2 and Supplementary Table S2, statistical analysis was applied to discover significant differences among sample categories, including sampling site and sediment layer. The results showed the mean sediment moisture of site 3 was lower than those of sites 1 and 6 in winter (Kruskal-Wallis test, adjusted *p*<0.05). The average pH at site 5 was lower than at sites 1 and 6 in either summer or winter (adjusted *p*<0.0419), and the average pH at site 3 was significantly lower than at site 1 in winter (adjusted *p*=0.0473). In summer, the sulfate concentrations at site 6 were lower than those at sites 3 and 5 (adjusted *p*<0.05). These results agreed with the previous observation that sites 3 and 5 shared a common physicochemical profile, which differed from those at sites 1 and 6. Moreover, differences among the physicochemical profiles of samples from different depth intervals were not significant (Kruskal-Wallis test, approximate *p*>0.05).

### Spatial Dynamics of Archaea Abundance and Diversity

The archaeal abundance in the Huixian karst wetland was evaluated *via* the archaeal 16S rRNA gene abundance (Figure 2), which ranged from 5.3E+04 to 2.7E+10 gene copies/g sediment. As shown in Figure 2, at sites 1, 5, and 6, the vertical variation of archaeal 16S rRNA gene abundances showed no clear trends, and among these three sampling sites, the variation of archaeal 16S rRNA gene abundances was low.

The vertical distribution pattern of the 16S abundance in site 3 was different from those of sites 1, 5, and 6. Site 3 had a broader abundance range than did the other sites, which tended to decrease from the top layers to the second bottom layers of the sediment in both summer and winter. Meanwhile, the 20–25-cm-deep samples (s3d5 and w3d5) from site 3 had the lowest copy number of 5.3E+04 gene copies/g sediment in the summer and 1.5E+06 copies/g sediment in the winter.

A Kruskal-Wallis one-way analysis showed the 16S rRNA gene abundance of site 3 was lower than that of site 1 (adjusted *p*=0.0197) in summer, and lower than those of either site 1 (adjusted *p*=0.0197) or site 5 (adjusted *p*=0.0076) in winter. Additionally, at each site no 16S-rRNA-gene-abundance difference was found among samples from different depth intervals (approximate *p*>0.05).

**Figure 3:**
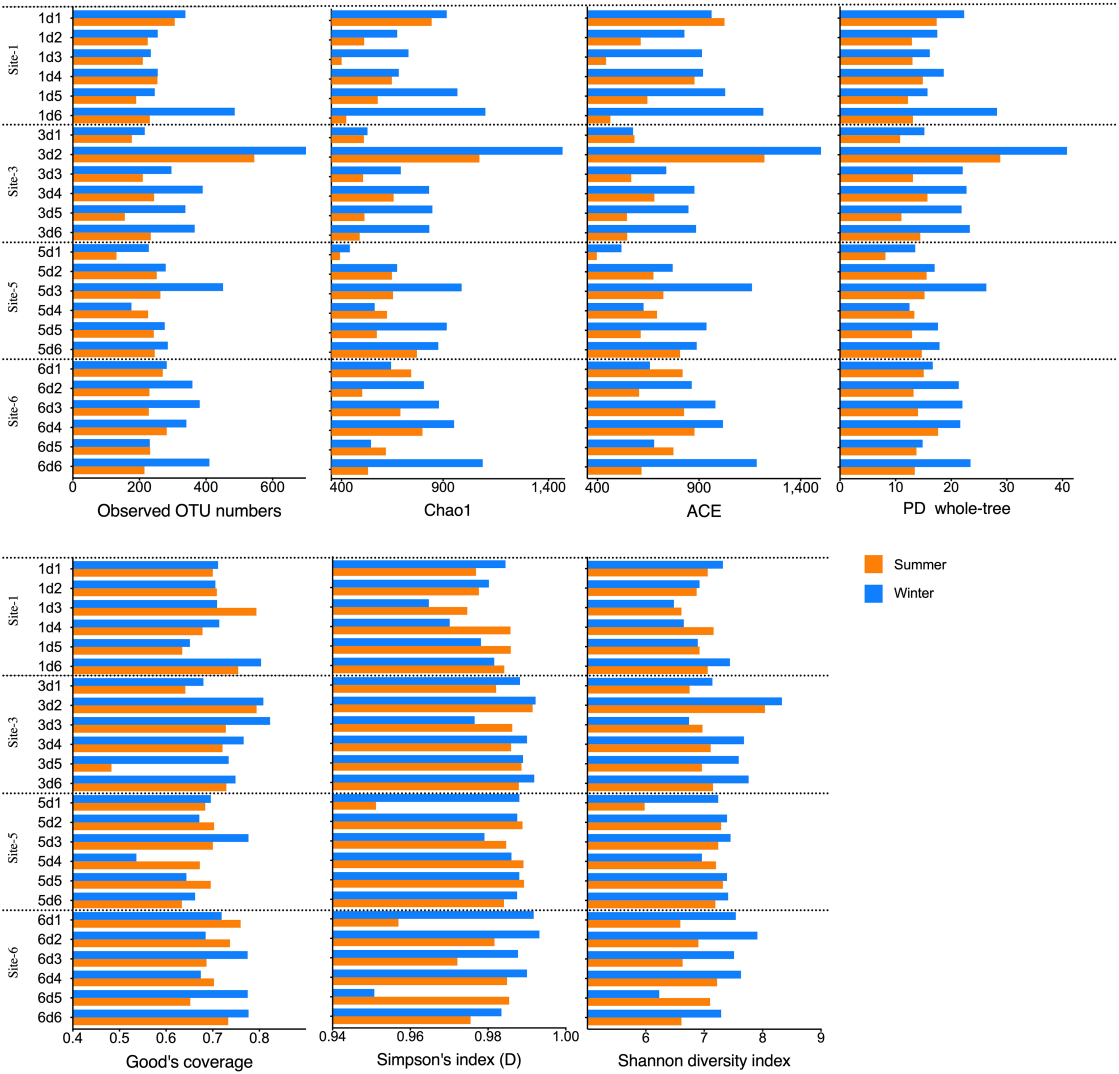
Alpha diversity indices (Observed OTU numbers, Chao1, ACE, PD whole-tree, Shannon and Simpson indices) of the sediment samples.

The alpha diversity indices, including the OTUs, Chao1, ACE, PD whole-tree indices, Good’s coverage, and the Shannon and Simpson indices, were calculated to explore the archaeal community richness and diversity (Figure 3; Supplementary Table S3). In total, 2,510 OTUs with 97% similarity were estimated from the 48 summer and winter samples. Each sample contained 131–724 OTUs (mean, 284 OTUs). When calculating the richness *via* Chao1 and ACE, the Chao1 index ranged from 392 to 1,488 (mean value 393), and the ACE ranged from 396 to 1,589 (mean value 397). The Good’s coverage of the archaeal communities ranged from 48 to 82% and the average value was 71%. The highest OTU and Chao1 and ACE values occurred in samples s3d2 and w3d2, both from site 3 in the 5–10-cm layer. For samples from the same season, the alpha diversity indices among the four sampling sites or from different depth intervals did not significantly differ (Kruskal-Wallis test, approximately *p*>0.05).

### Spatial Variations of Archaeal Community Structure

Archaeal communities in the four studied sites were mainly composed of two dominant phyla, Euryarchaeota (57.4%) and Bathyarchaeota (38.7%), and four minority phyla, Thaumarchaeota (2.0%), Woesearchaeota (0.9%), Crenarchaeota (0.4%), and Diapherotrites (0.2%). Euryarchaeota was mainly composed of five groups of archaea, which were methanogenic archaea of the order of Methanosarcinales (25.1%), Methanomicrobiales (13.7%), Methanomassiliicoccales (4.6%) and Methanocellales (3.8%), and anaerobic methanotrophic archaea ANME-2d (9.0%). Bathyarchaeota was dominated by subgroups MCG-11, MCG-6, and MCG-5b, which contributed 16.5, 9.1, and 5.5% of the archaeal communities, respectively.

**Figure 4:**
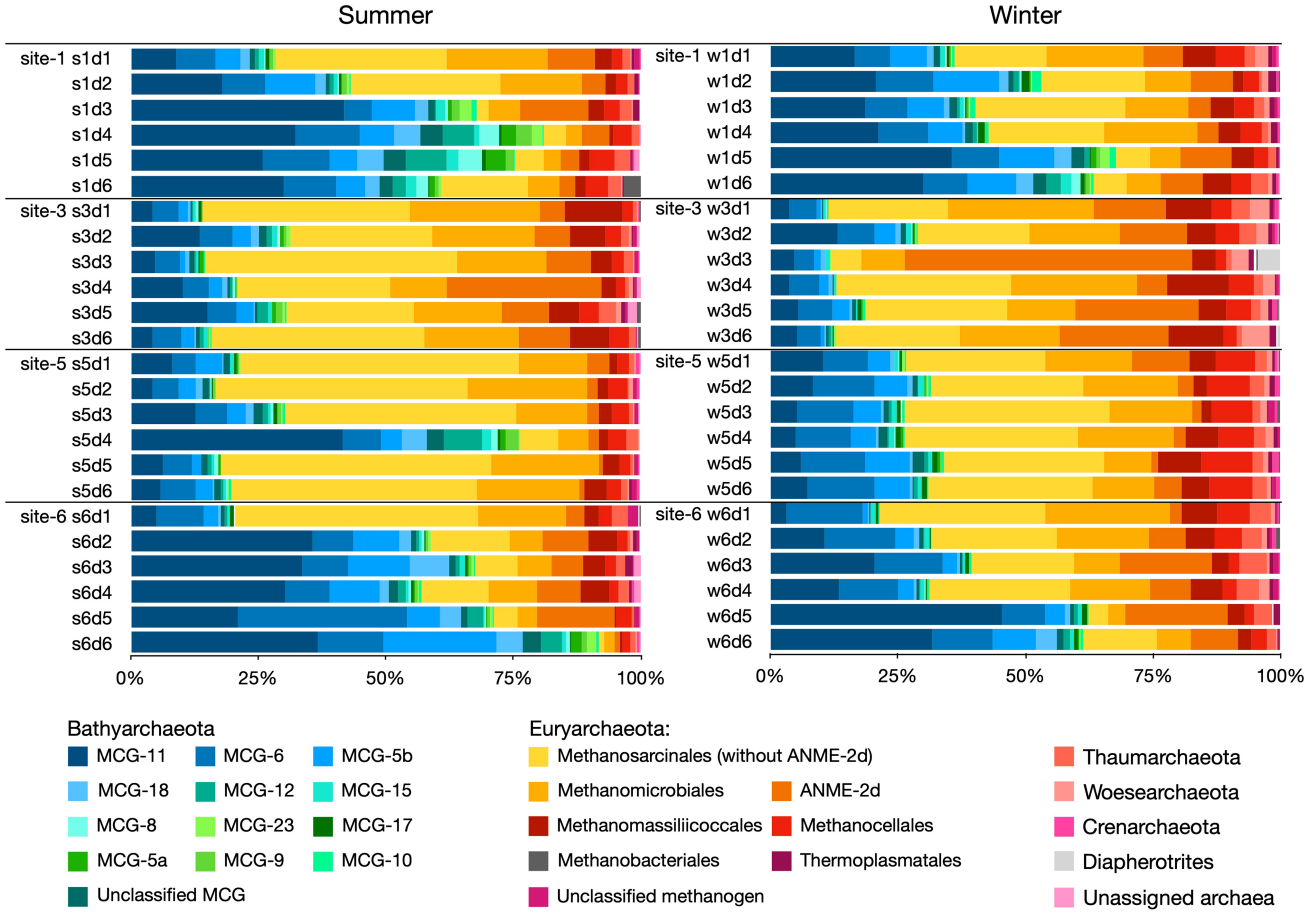
Archaeal taxonomic community compositions in different samples as revealed by 16S rRNA gene sequencing.

At the archaeal phylum level, the vertical distributions of Euryarchaeota and Bathyarchaeota conformed to two patterns among the four sampling sites (Figure 4). “Pattern A” was designated for sampling sites 1 and 6, where the Bathyarchaeota tended to dwell in deep layers of the sediment and Euryarchaeota were commonly found in the surface sediment. “Pattern B” was proposed to describe the vertical distribution of archaeal phyla at sites 3 and 5, where the vertical variation of Euryarchaeota and Bathyarchaeota was low, Euryarchaeota dominated almost all the layers of sediment (except s5d4), and their relative abundance remained the same as depth increased. At the order/subgroup level, showing the distribution profiles that Methanosarcinales, Methanomicrobiales, and MCG-11 were consistent with patterns A and B, except for samples s3d4 and w3d3, where the ANME-2d archaea were found to be the dominant subgroup over methanogenic Methanosarcinales and Methanomicrobiales.

Indicator species analyses were applied to reveal the site-preferences of archaeal order/subgroup. As shown in Table 1, the results showed that sites 1 and 6 shared two common indicator archaea (MCG-11 and MCG5b), suggesting that they were commonly found at site 1 and site 6; site 3, and site 5 also had two common indicator archaea (methanogenic Methanosarcinales and Methanomicrobiales). In addition, the indicator species of the single sampling site 3 were ANME-2d and Methanomassiliicoccales; for site 5 it was Methanocellales; for site 6, it was MCG-6.

**Table 1:** Differentially abundant archaeal orders, subgroups, and phyla (relative abundance ≥1%) associated with selected classification groups based on an indicator species analysis.

Classification	Taxon (phylum, order, and subgroup)	IndVal^*^	Value of *p*	Significance codes
Site 3	Methanomassiliicoccales	0.564	0.0001	***
	ANME-2d	0.497	0.0006	***
Site 5	Methanocellales	0.503	0.0032	**
Site 6	MCG-6	0.48	7.00E-04	***
Site 1+Site 6	MCG-11	0.634	0.0001	***
	MCG-5b	0.508	0.001	***
Site 3+Site 5	Methanogenic Methanosarcinales	0.558	0.0005	***
	Methanomicrobiales	0.5	0.0025	**
0–10-cm-deep sediment	Methanomicrobiales	0.481	0.0127	*
Winter	Methanocellales	0.505	0.0002	***
	Thaumarchaeota	0.421	0.0026	**
	Methanomassiliicoccales	0.361	0.0126	*

* IndVal: statistical value of indicator species analysis; a higher value means that the OTU is more strongly associated. ^*^p<0.05; ^**^p<0.01; ^***^p<0.001.

**Figure 5:**
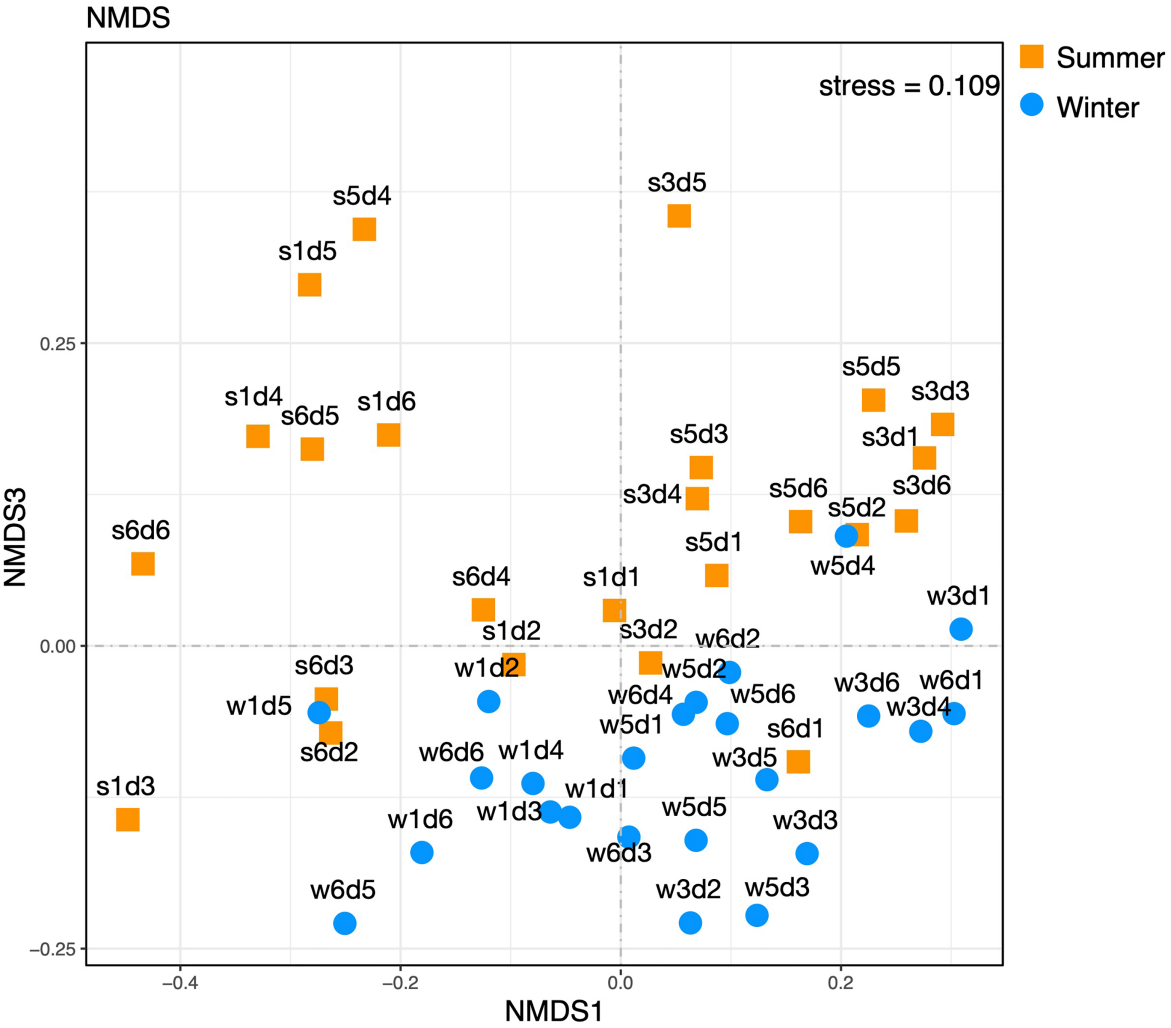
Non-metric multidimensional scaling plot of 3% OTU compositions from the summer and winter samples.

At the OTU level of archaeal composition, statistical differences among the samples from different sites per the ANOSIM test were evident. Grouping the samples by sampling site revealed statistical differences (ANOSIM for site: *r*=0.3001, *p*=1e^−4^). However, the Bray-Curtis non-metric multidimensional scaling (NMDS, as shown in Figure 5) results showed no obvious differences among samples from different sampling sites and depths at OTU levels of archaeal composition.

In terms of depth, the indicator species analysis results showed that Methanomicrobiales was the only indicator associated with sediment depth, which was 0–10cm (Table 1). An ANOSIM test of OTU-level archaeal composition revealed no statistically significant differences when samples were grouped by depth.

### Changes in Biophysiochemical Profiles Between Summer and Winter

The most observed temporal differences in the physicochemical properties were in the surface sediment temperature of all the sampling sites and the nitrate concentration in each sample. The temperatures of each of the four sampling sites were consistent during the summer (~28°C) and winter (~20°C). Nitrate was detected in all the summer samples with a concentration of 0.1–1.7mg/L pore water. However, nitrate was observed in only four winter samples (w1d3, w5d2, w6d3, and w6d5) and was either absent or too low to detect in the remaining samples. In addition, the mean pH of the winter samples was significantly lower than that of summer samples (Mann–Whitney test, *p*=0.003), which were 7.407 and 7.505, respectively. Additionally, the sulfate concentrations in the sediment pore water had a broader range in winter (12.14–74.5mg/l) than in summer (0.45–9.95mg/l). Despite these differences, samples from summer and winter showed no significant differences in moisture content, dissolved calcium ions, and sulfate concentration (*p*>0.05).

The archaeal community abundance was significantly higher in the winter samples than in the summer samples (Mann–Whitney test, *p*<0.0001). In the winter samples, the average archaeal 16S rRNA gene copy number was 1.0E+10 per gram wet sediment; in the summer samples, the mean copy number was 2.6E+07 per g wet sediment. Temporal significant differences also existed between the samples from the same site (Mann–Whitney test, *p*=0.0022) and in samples from the same depth intervals (Mann–Whitney test, *p*=0.0286) with one exception; no significant temporal difference was found for the samples from the 25–30-cm depth interval (Mann–Whitney test, *p*=0.2).

Statistical comparisons of the alpha diversity indices among samples revealed that samples taken in winter generally had higher alpha diversity indices than the summer samples (Mann–Whitney test, *p*<0.05). For all the winter samples, the OTU numbers, Chao1, PD whole-tree, ACE and Shannon indices were higher than those of the summer samples per the Mann–Whitney test (*p*≤0.0072). At site 1, the Chao1 (*p*=0.0152) and PD whole-tree (*p*=0.0087) indices were higher in the winter samples than in the summer samples. At site 6, the winter samples shower higher OTUs (*p*=0.00173) and PD whole-tree (*p*=0.0152) indices than the summer samples. Winter samples from 0 to 5 cm sediment depth showed higher Shannon and Simpson indices (*p*=0.0286) than the summer samples. The winter samples from 25 to 30cm depths showed higher ACE, Chao1, and PD whole-tree indices (p=0.0286) than the summer samples, and the winter samples from 10 to 15 cm depths had higher Chao1 and PD whole-tree indices (*p*=0.0286) than the summer samples.

### Variations of the Archaeal Community Composition Between Summer and Winter

The relative abundance of archaeal phyla (with abundance >1%) in the summer and winter samples was not significantly different. From summer to winter, the mean relative abundance of Euryarchaeota increased from 54.3 to 60.5% (Mann–Whitney test *p*=0.7054), for Bathyarchaeota, it decreased from 43.2 to 34.3% (*p*=0.3815), and for Thaumarchaeota, it increased from 1.5 to 2.5% (*p*=0.0028). The Mann–Whitney test suggested that only the relative abundance of Thaumarchaeota varied significantly between summer and winter as evidenced by the values of *p* less than 0.05. This phenomenon was also supported by an indicator species analysis (Table 1), which showed that members of Thaumarchaeota were more commonly found in the winter samples than in the summer samples.

At the order/subgroup level, only the relative abundance of Methanocellales and Methanomassiliicoccales were found to be higher in the winter samples than in the summer samples per the Mann–Whitney test (*p*=0.0009 and 0.0066) and an indicator species analysis (Table 1). Analyses of samples from specific site or depth intervals, revealed significant temporal differences in relative abundance. At site 1, Methanomassiliicoccales showed a higher relative abundance in the winter samples than in the summer samples per the Mann–Whitney test (*p*=0.0411); at site 3, Methanosarcinales was more abundant in the summer samples than in the winter samples (p=0.0411); at site 5, the relative abundance of Methanocellales, Methanomassiliicoccales, MCG-6, and MCG-5b was higher in the winter samples than in the summer samples (*p*=0.0022, 0.0455, 0. 0022, and 0.00871, respectively); and within the 15–20cm depth interval, Methanomicrobales was more abundant in the winter samples than in the summer samples (*p*=0.0286).

At the OTU level, statistical differences among the samples from different seasons per ANOSIM test (*r*=0.1043, *p*=0.0098) and NMDS results (Figure 5) were seen, which showed plots representing winter and summer samples were not clearly separated, although differences existed.

### Relationship Between Community Properties and Physicochemical Parameters

A Spearman correlation analysis was performed to determine the relationship between the physicochemical parameters and the alpha diversity indices (Table 2). The observed OTU numbers, the Chao1, ACE, and PD whole-tree indexes were negatively correlated with the nitrate concentration in the sediment; the Simpson and Shannon indices were positively correlated with the sulfate concentration but negatively correlated with the pH and the moisture content, and the Shannon index was positively correlated with the Ca^2+^ concentration.

**Table 2:** Spearman correlation analysis between physicochemical parameters and alpha diversity indices, major euryarchaeotal orders, bathyarchaeotal subgroup proportions and 16S rRNA gene abundances.

	Nitrate	Sulfate	pH	Moisture content	Depth	Ca^2+^
Observed OTUs	−0.434^**^	0.095	−0.185	−0.121	0.033	0.253
Chao1	−0.473^***^	−0.065	−0.173	−0.074	0.126	0.128
ACE	−0.448^**^	−0.166	−0.061	0.057	0.107	0.188
PD whole-tree	−0.550^***^	−0.025	−0.223	−0.079	0.03	0.18
Good’s coverage	−0.196	−0.014	0.091	0.067	−0.031	−0.019
Simpson’s index (D)	−0.109	0.510^***^	−0.322^*^	−0.485^***^	0.047	0.163
Shannon diversity index	−0.283	0.375^**^	−0.364^*^	−0.362^*^	0.079	0.29^*^
MCG-11	0.153	−0.311^*^	0.408^**^	0.453^**^	0.269	0.129
MCG-6	−0.247	−0.087	0.198	0.355^*^	0.16	0.437^**^
MCG-5b	−0.118	−0.714^***^	0.355^*^	0.455^**^	0.132	0.108
Methanosarcinales (without ANME-2d)	0.167	0.306^*^	−0.351^*^	−0.428^**^	−0.266	−0.136
Methanomicrobiales	−0.097	0.25	−0.317^*^	−0.258	−0.417^**^	−0.047
ANME-2d	−0.225	−0.044	0.238	0.094	0.006	−0.075
Methanomassiliicoccales	−0.493^***^	0.092	−0.133	−0.056	−0.068	−0.134
Methanocellales	−0.411^**^	0.034	−0.284	−0.167	−0.022	0.119
16S rRNA gene copies/g wet sediment	−0.538^***^	−0.092	−0.332^*^	0.356^*^	0.112	0.084

* *p<0.05*;

** *p<0.01*;

*** *p<0.001*.

In the sediment, the archaeal 16S rRNA gene abundance was positively correlated with the moisture content and negatively correlated with nitrate (*p*<0.001) and pH (*p*<0.05; Table 2). Regarding the correlation between physicochemical parameters and the dominant archaeal groups, the proportions of both MCG-11 and MCG-5b were positively correlated with pH and the moisture content and negatively correlated with sulfate. MCG-6 was positively correlated with the moisture content and the Ca^2+^ concentration in the sediment; Methanosarcinales (excluding ANME-2d) was negatively correlated with the moisture content and pH, and Methanomicrobiales was negatively correlated with depth and pH per a Spearman correlation analysis.

**Figure 6:**
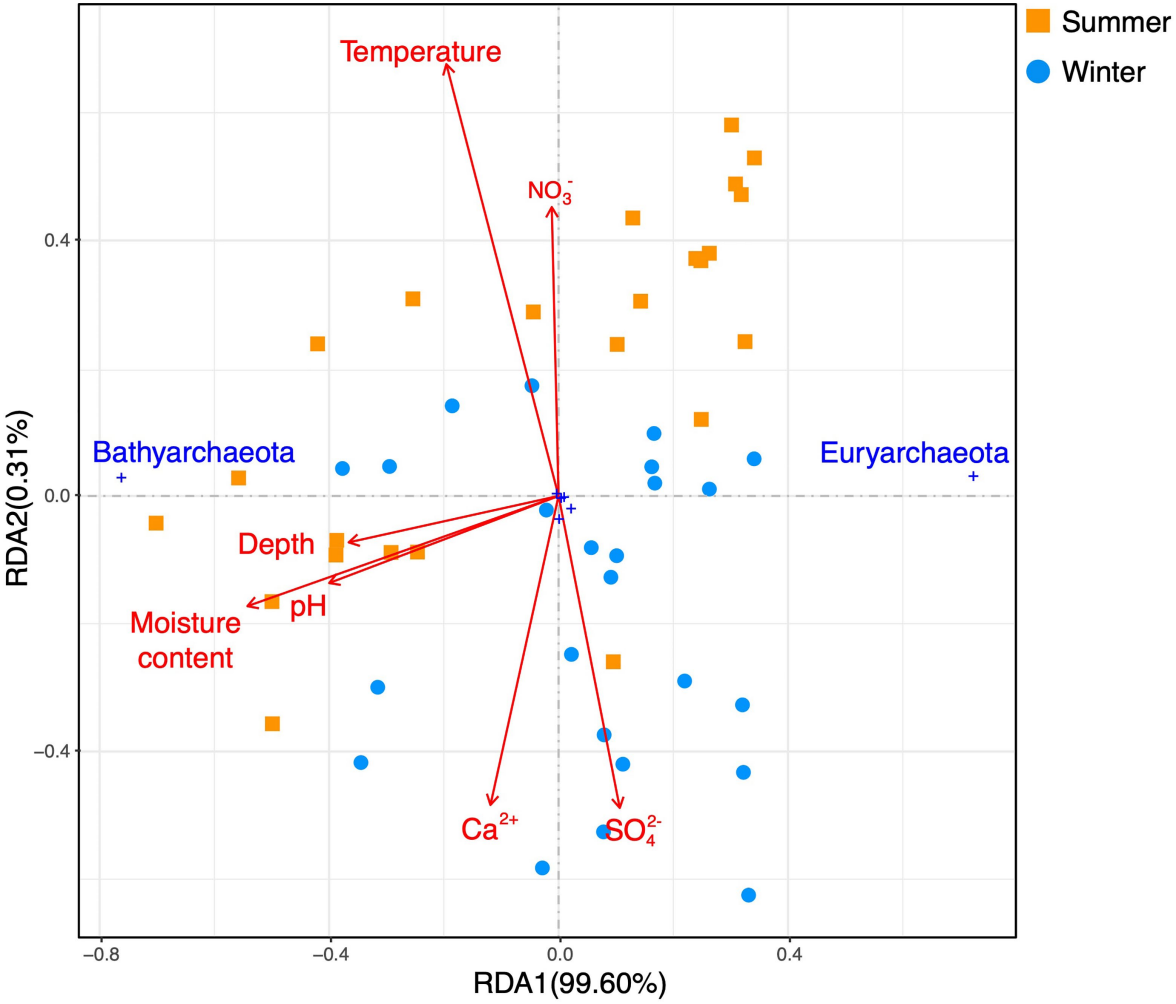
A redundancy analysis based on environmental variables (pH, depth, moisture content, surface sediment temperature, and dissolved Ca^2+^, sulfate, and nitrate in the sediment pore water) fitted to the proportions of archaeal phyla.

**Figure 7:**
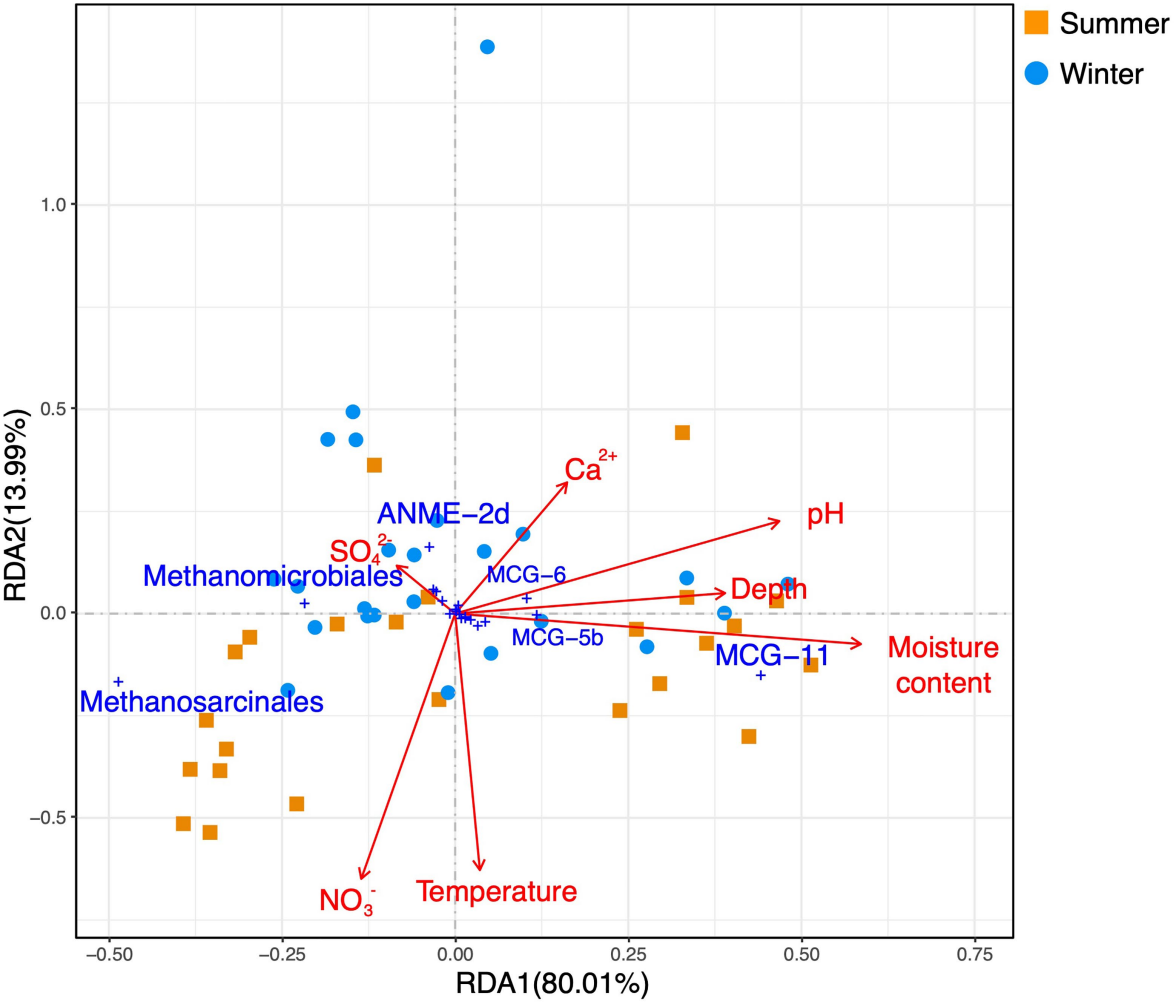
A redundancy analysis based on environmental variables (pH, depth, moisture content, surface sediment temperature, and dissolved Ca^2+^, sulfate, and nitrate in the sediment pore water) fitted to proportions of euryarchaeotal orders and bathyarchaeotal subgroups.

A redundancy analysis (RDA) based on phylum-level archaeal relative abundance indicated that the first two axes of the plots explained 99.6 and 0.31% of the variance of the whole archaeal community, respectively (Figure 6). The moisture content, pH, and depth were significantly correlated with Bathyarchaeota. A RDA based on euryarchaeotal orders and bathyarchaeotal subgroups (Figure 7) showed that the first two axes of the plots, respectively, explained 80.1 and 13.99% of the variance in the community structure, and the moisture content, pH, and depth were the most important factors affecting MCG-11.

## Discussion

Archaea play significant role in an environment; however, due to the complexity and diversity of karst environments, the studies on archaea in karst environments are scarce. A few previous studies (Nold et al., 2010; Fillol et al., 2015; Lazar et al., 2017; Davis and Garey, 2018; Hershey et al., 2018; Menning et al., 2018) reported that archaeal communities exist in different types of karst environments. Bathyarchaeota and Euryarchaeota were the dominant archaeal phyla in the sediment of the Huixian karst wetland, similar to those found in the lakes of the Banyoles karstic system (Banyoles, Spain; Fillol et al., 2015), and other wetlands (Ma et al., 2016; Li et al., 2017a). The 16S rRNA gene abundance and alpha diversity indices in the winter samples of the Huixian karst wetland were apparently higher than those of the Banyoles karstic system and other wetlands (Ma et al., 2016; Li et al., 2017a, 2019), but similar to those of mangrove and intertidal wetlands (Zhou et al., 2017). In addition to the findings described above, this work has shown the spatial–temporal variations of archaeal abundance, diversity, and composition. Environmental factors affecting these variations and niche preferences of specialized archaea are discussed below:

### Factors Driving the Variation of Archaeal Composition, Abundance, and Diversity

#### Moisture Content

Soil moisture content is thought to be a significant environmental factor regulating oxygen diffusion and affecting the archaeal community structure and diversity (Stres et al., 2008; Li et al., 2019). Previous studies (Li et al., 2019) of the archaeal communities in the East Dongting Lake wetland shown the relative abundance of Euryarchaeota and Methanomicrobiales increased with the soil moisture content. However, in this work the correlation between Methanomicrobiales and the sediment moisture content was not significant. The results presented in Table 2 show that the methanogenic Methanosarcinales were negatively correlated with the moisture content, and all three dominant bathyarchaeotal subgroups (MCG-11, MCG-6, and MCG-5b) were positively correlated with the moisture content.

The correlation between the moisture content and archaea leads to the two patterns of archaeal vertical distribution among the four sampling sites. In 0–30cm sediment at each sampling site, the vertical distribution of archaea conformed to two patterns (Pattern A and Pattern B). In Pattern A at sites 1 and 6, Euryarchaeota tended to exist in the surface layers of the sediment, while Bathyarchaeota usually existed in the bottom layers of the sediment. In Pattern B at sites 3 and 5, Euryarchaeota dominated almost all of the sediment layers (except s5d4), and the relative abundance did not change with increasing depth. These phenomena did not likely result from the water depth gradients of the four sampling sites or the macrophyte inhabitants, because the water depths at site 1 and site 6 were distinct (1m at site 1 and 3cm at site 6), but shared the same archaeal distribution pattern; the archaeal vertical distribution Pattern B was observed at sites 3 and 5, and the water depths at sites 3 and 5 were 70 and 50cm, respectively, while site 3 had macrophyte inhabitants. The vertical archaeal distribution patterns might be determined by the moisture content at each sampling site. As shown in Figure 2 and Supplementary Table S2, from 0 to 30 cm deep, the moisture content for sites 1 and 6 was comparatively constant at ~50%; however, at sites 3 and 5, the moisture content at 5–30cm deep was approximately 35%. The mean moisture content at sites 3 and 5 was, respectively, 40.18 and 38.13% in the summer and 38.67 and 40.10% in the winter, lower than those at sites 1 and 6 (respectively 50.3 and 50.8% in the summer, and 53.41 and 52.84% in the winter). These differences were partially supported by a Kruskal-Wallis test, which showed that the moisture content difference between winter samples from site 1 and site 3 was significant (*p*<0.0225).

#### pH

Soil pH is a prevailing environmental factor that shapes the biogeographic patterns of archaea. In this work, in addition to the moisture content, the sediment pH may contribute to the shape of the archaeal vertical distribution. The mean pH values for sites 1 and 6 were, respectively, 7.79 and 7.72 in the summer and 7.52 and 7.49 in the winter, which was higher than at sites 3 and 5 (respectively 7.60 and 6.92 in the summer and 7.34 and 7.29 in the winter). As previously mentioned, the pH differences between site 5 and site 1 (or between site 5 and site 6) were significant in either summer or winter (Kruskal-Wallis test, *p*<0.0419). Additionally, the pH was found to be negatively correlated with both methanogenic Methanosarcinales and Methanomicrobiales, and positively correlated with MCG-11 and MCG-5b (Table 2; Figure 7). Therefore, pH likely affected the archaeal biogeographic patterns by influencing the relative abundance of the dominant archaeal subgroups in the Huixian karst wetland.

The correlation between sediment pH and the two methanogenic archaeal orders was similar to previous reports, in which the relative abundance of Methanomicrobiales was negatively correlated with soil pH in paddy soil (Yuan et al., 2019) and lake sediment (Li et al., 2019). However, the correlations between pH and bathyarchaeotal subgroups (MCG-11 and 5b) have not been reported previously to this study.

#### Nitrate and Temperature

The present results showed the most notable temporal variations in archaea were the differences in the archaeal 16S rRNA gene abundance and diversity between the summer and winter samples. All the winter samples showed higher 16S rRNA gene abundance and Chao1, ACE, and PD whole-tree indexes than did the corresponding summer samples, possibly due to a combined effect of various temporal factors. Similar to these results, a significant decline in archaeal abundance has been observed in the summer samples of a mesocosm wetland and was considered a result of high temperature (Li et al., 2017a). In the present study, in addition to temperature, the occurrence of nitrate in all summer sediment samples is a likely reason for the observed difference in 16S RNA gene abundance, because the presence of nitrate may increase the redox potential of the sediment to positive levels, resulting in unfavorable conditions for methanogens, which require a redox potential of −200 to −400mV (Fetzer and Conrad, 1993; Clarens et al., 1998; Hirano et al., 2013), thus leading to a significant decrease in methanogenic archaeal abundance. A Spearman correlation analysis (Table 2) showed that the nitrate concentration was significantly negatively correlated with the proportion of Methanomassiliicoccales and Methanocellales. The reduction in methanogens may also have decreased the abundance and diversity of ANME-2d and Bathyarchaeota because ANME-2d live on methane, and Bathyarchaeota are thought to form a symbiotic relationship with the methanogenic archaea (Xiang et al., 2017).

#### Other Factors

Site 3 was unique for its lower average archaeal 16S-rRNA-gene-abundance, and higher mean relative abundance of ANME-2d and Methanomassiliicoccales archaea, compared to the other three sites. Meanwhile, the highest OTU and Chao1 and ACE values were observed in the 5–10-cm- layer samples of site 3. Although sites 3 and 5 shared more common physicochemical profiles than with sites 1 and 6, site 3 was the only sampling site located in an artificial pond and with macrophyte inhabitants. These features may have resulted in the unique characteristics of the archaeal abundance and diversity in the sediment of site 3.

The sulfate concentration in the sediment pore water was negatively correlated with the relative abundance of MCG-11 and MCG-5b (Table 2); however, these correlations were not strong according to the RDA results (Figure 7). The sulfate concentration in the sediment of sites 1, 3, and 5 showed an increasing trend with an increase of sediment depth, which indicated the presence of sulfate reducers in the surface layers of the sediment.

Calcium-richness is one of the water properties in the karst environment. To date, the impact of calcium ions on the archaea remains unclear. The results presented (Table 2; Figure 7) suggested that dissolved calcium ions in the sediment were positively correlated with the relative abundance of MCG-6.

The water level of the pond affects the available nutrients in the sediment. The samples studied here were obtained from four sampling sites in ponds of the Huixian karst wetland. These sites had different water depths. Sample site 6 had the lowest depth (3cm), which may have resulted in significantly higher concentrations of sulfate and calcium in the surface layers of the sediment in winter. Besides that, no correlation between the water depth and archaeal communities was found (Spearman correlation test *p*>0.05).

### Niche Preferences of Specialized Archaea in the Huixian Karst Wetland

#### ANME-2d Archaea

The ANME archaea, together with denitrifying methane-oxidizing bacteria from the candidate phylum of NC10 are capable of AOM (Ettwig et al., 2010). AOM is considered as a significant sink for methane, which plays important role in global warming. This process occurs in both marine and freshwater environments. In marine environments, AOM has been well documented, where ANME were reported to be abundant in anoxic marine environments, consuming up to 90% of the methane produced from the sediment (Knittel and Boetius, 2009). In comparison, AOM was only recently recognized as an important sink for methane in freshwater environments such as lakes and peatlands (Segarra et al., 2015; Li et al., 2017a). Scarce data are available on niche preferences and the taxonomic identification of freshwater AOM-mediating microorganisms.

In this study, one group of the ANME population, the ANME-2d cluster archaea, was identified as the third most abundant Euryarchaeota subgroup. Approximately 140 ANME-2d OTUs were found; these sequences were restricted to the ANME-2d branch as shown in Supplementary Figure S1. Some of the sequences may represent novel species of ANME-2d. A phylogenetic analysis of these OTUs suggests some of the ANME-2d members likely represented species common across various environments, such as petroleum-contaminated soil, iron-rich freshwater, and acidic peatlands, because their sequences were in the same subbranches as the reference sequences. A considerable amount of ANME-2d OTUs formed independent subbranches distinct from the reference sequences, which may be unique to karst wetlands. These findings extend our knowledge of the diversity of the ANME-2d archaea.

ANME-2d was commonly found in the samples from site 3 by an indicator species analysis. Site 3 is the only sampling site, where macrophytes were found. These results suggested that the presence of macrophytes may favor the growth of ANME and shifts in the dominant macrophytes may not affect the proportion of ANME-2d in archaeal communities. In winter, site 3 was the only site with submerged green plants, and in summer, the surface water was covered with water hyacinths; correspondingly, the mean relative abundance of ANME-2d decreased from 22.28 to 11.65%. However, a Mann–Whitney test suggested that this change was not significant (*p*=0.1797). Therefore, differences of the dominant macrophytes from submerged green plants to water hyacinths may not affect the proportion of ANME-2d in archaeal communities in this environment.

The conspicuous abundance of ANME-2d cluster archaea (9%) in the archaeal communities in this study suggests ANME-2d may have a role in reducing methane emissions from karst wetlands. The ANME-2d cluster archaea can couple anaerobic methane oxidation (AOM) to the reduction of various electron acceptors, including nitrate (Haroon et al., 2013), iron (Ettwig et al., 2016), sulfate (Schubert et al., 2011; Timmers et al., 2016), and humic substances (Valenzuela et al., 2017, 2019; Bai et al., 2019). Occurrences of nitrate and sulfate gradients in the sediment indicate that AOM in the Huixian karst wetland may be coupled with the reduction of nitrate or sulfate.

#### MCG-11, MCG-5b, and MCG-6 Bathyarchaeotal Subgroups

MCG-11 and MCG-5b were the first and third most dominant bathyarchaeotal subgroups identified in the present study and are considered to adapt well in freshwater sediment (Fillol et al., 2016; Xiang et al., 2017). The physiological functions and environmental factors affecting MCG-11 and MCG-5b are poorly understood. In this study, a Spearman correlation analysis showed that the proportions of both MCG-11 and MCG-5b were positively correlated with the moisture content and pH. RDA results suggested that MCG-11 was positively correlated with moisture content, pH, and depth. Furthermore, the proportion of MCG-11 was positively correlated with MCG-5b (Spearman *r*=0.608, *p*<0.001). These results indicate that both MCG-11 and MCG-5b prefer slightly alkalescent sediment with high moisture content. Alkalescence is one of the geochemical features of the karst environment, which may benefit the abundance of MCG-11 and MCG-5b.

MCG-6 was the second largest bathyarchaeotal subgroup identified in this research. It was the indicator of site 6 and was one of only three bathyarchaeotal subgroups (together with MCG-17 and MCG-10) to show a higher relative abundance in winter than in summer. Previous research on MCG-6 in a mangrove wetland suggested that MCG-6 adapts well in slightly acidic environments (Zhou et al., 2017). The average pH in the winter samples was lower than that in the summer samples (Mann–Whitney *p*=0.003), which may explain the higher relative abundance of MCG-6 in the winter than in the summer. Moreover, the results presented (Table 2; Figure 7) suggested MCG-6 may prefer a calcium-rich environment.

## Conclusion

We comprehensively investigated the archaeal community abundance, diversity, and composition at four sites in the Huixian karst wetland in summer and winter. The results presented suggest that these karst wetlands shared a similar phylum-level archaeal composition with those of karstic lakes and other freshwater wetlands. The archaeal community composition remained stable, but their abundance and diversity varied seasonally. In winter, the archaeal abundance and diversity were higher than in other karst systems and wetlands, but in summer it showed an opposite trend. The nitrate concentration in the sediment was probably the factor affecting the archaeal abundance and diversity, and moisture content and pH were the factors that most affected the spatial variation of the archaeal communities. Additionally, the common features of karst environments, calcium-richness and weak alkalescence of the water supply may favor the abundance of the bathyarchaeotal subgroups MCG-11, −5b and MCG-6. Identification of methanogens, methanotrophic archaea, and subgroups of Bathyarchaeota suggests that archaea may contribute greatly to the carbon cycle because of their involvement in methanogenesis, anerobic methane oxidation, and organic component degradation. This capability remains stable across seasons, and archaeal activity is significantly influenced by the input of nitrate from human activities.

## Data Availability Statement

The PacBio Reads of the Insert data were deposited in GenBank as Sequence Read Archive PRJNA686692.

## Author Contributions

YC conceived this study, performed the data analysis, and wrote the manuscript. YC, KQ, ZZ, and TZ collectively contributed to the sampling, molecular biology experiments, and some of the physicochemical analyses. All authors contributed to the article and approved the submitted version.

## Conflict of Interest

The authors declare that the research was conducted in the absence of any commercial or financial relationships that could be construed as a potential conflict of interest.

## Publisher’s Note

All claims expressed in this article are solely those of the authors and do not necessarily represent those of their affiliated organizations, or those of the publisher, the editors and the reviewers. Any product that may be evaluated in this article, or claim that may be made by its manufacturer, is not guaranteed or endorsed by the publisher.

## Footnotes

^1^ www.geneious.com

^2^ https://www.arb-silva.de

^3^ www.graphpad.com

^4^ https://www.omicshare.com/tools/home/index/index.html
