# Supplementary material for: Influence of Environmental Factors on the Variability of Archaeal Communities in a Karst Wetland
Source: Front Microbiol. 2021 Sep 3;12:675665. doi: 10.3389/fmicb.2021.675665 (PMC8448418; doi:10.3389/fmicb.2021.675665)
Supplement: Supplementary file 1 [file Data_Sheet_1.docx]

Supplementary Material

# This document contains:

# 1 Supplementary table

Table S1

Table S2

Table S3

# 2 Supplementary figures

Supplementary Figure S1

**1 Supplementary table**

# Table S1 Barcode sequences and corresponding sample names

| Summer sample | Winter sample | Barcode sequences |
| --- | --- | --- |
| s1d1 | w1d1 | TCCCTTGTCTCC |
| s1d2 | w1d2 | ACGAGACTGATT |
| s1d3 | w1d3 | GCTGTACGGATT |
| s1d4 | w1d4 | ATCACCAGGTGT |
| s1d5 | w1d5 | TGGTCAACGATA |
| s1d6 | w1d6 | ATCGCACAGTAA |
| s3d1 | w3d1 | GTCGTGTAGCCT |
| s3d2 | w3d2 | AGCGGAGGTTAG |
| s3d3 | w3d3 | ATCCTTTGGTTC |
| s3d4 | w3d4 | TACAGCGCATAC |
| s3d5 | w3d5 | ACCGGTATGTAC |
| s3d6 | w3d6 | AATTGTGTCGGA |
| s5d1 | w5d1 | TGCATACACTGG |
| s5d2 | w5d2 | AGTCGAACGAGG |
| s5d3 | w5d3 | ACCAGTGACTCA |
| s5d4 | w5d4 | GTAGATCGTGTA |
| s5d5 | w5d5 | TAACGTGTGTGC |
| s5d6 | w5d6 | CATTATGGCGTG |
| s6d1 | w6d1 | CCAATACGCCTG |
| s6d2 | w6d2 | GAATACCAAGTC |
| s6d3 | w6d3 | GCGCGATACGATGACT |
| s6d4 | w6d4 | CATAGCGACTATCGTG |
| s6d5 | w6d5 | GCTCGACTGTGAGAGA |
| s6d6 | w6d6 | ACTCTCGCTCTGTAGA |

**Table S2 Physicochemical parameters of samples collected from the Huixian karst wetland in the summer and winter.**

| Samples | Sedient depth (cm) | pH | Nitrate  (mg/L) | Sulfate (mg/L) | Moisture content (%) | Ca 2+  (mg/L) | Surface water depth (m) | Temperature (℃) | Macrophyte inhibiting |
| --- | --- | --- | --- | --- | --- | --- | --- | --- | --- |
| s1d1 | 0-5 | 7.78 | 0.54 | 1.09 | 51.01 | 128.43 | 1 | 28 | NO |
| s1d2 | 5-10 | 7.81 | 0.28 | 1.27 | 50.99 | 159.31 | 1 | NA | NO |
| s1d3 | 10-15 | 7.92 | 0.52 | 0.92 | 54.15 | 134.25 | 1 | NA | NO |
| s1d4 | 15-20 | 7.93 | 0.62 | 3.20 | 47.95 | 138.97 | 1 | NA | NO |
| s1d5 | 20-25 | 7.75 | 0.49 | 3.40 | 48.97 | 125.26 | 1 | NA | NO |
| s1d6 | 25-30 | 7.53 | 0.50 | 4.99 | 48.75 | 112.73 | 1 | NA | NO |
| s3d1 | 0-5 | 7.48 | 0.29 | 1.42 | 60.63 | 104.91 | 0.7 | 28 | water hyacinth |
| s3d2 | 5-10 | 7.55 | 0.35 | 1.82 | 40.04 | 117.32 | 0.7 | NA |  |
| s3d3 | 10-15 | 7.52 | 0.61 | 4.64 | 35.57 | 119.27 | 0.7 | NA |  |
| s3d4 | 15-20 | 7.65 | 0.67 | 6.11 | 34.72 | 107.35 | 0.7 | NA |  |
| s3d5 | 20-25 | 7.66 | 0.85 | 5.70 | 32.99 | 105.23 | 0.7 | NA |  |
| s3d6 | 25-30 | 7.71 | 0.99 | 3.32 | 37.11 | 108.54 | 0.7 | NA |  |
| s5d1 | 0-5 | 7.50 | 1.06 | 1.27 | 49.71 | 108.73 | 0.5 | 28 | NO |
| s5d2 | 5-10 | 7.03 | 0.89 | 2.73 | 34.76 | 117.34 | 0.5 | NA | NO |
| s5d3 | 10-15 | 6.80 | 1.05 | 4.06 | 36.39 | 115.76 | 0.5 | NA | NO |
| s5d4 | 15-20 | 6.71 | 1.48 | 9.95 | 34.69 | 107.38 | 0.5 | NA | NO |
| s5d5 | 20-25 | 6.76 | 1.75 | 8.10 | 36.74 | 112.46 | 0.5 | NA | NO |
| s5d6 | 25-30 | 6.71 | 1.21 | 8.90 | 36.48 | 108.62 | 0.5 | NA | NO |
| s6d1 | 0-5 | 7.78 | 0.36 | 2.24 | 47.95 | 101.17 | 0.03 | 28 | NO |
| s6d2 | 5-10 | 7.77 | 0.15 | 0.65 | 51.91 | 103.71 | 0.03 | NA | NO |
| s6d3 | 10-15 | 7.83 | 0.37 | 0.50 | 44.66 | 116.42 | 0.03 | NA | NO |
| s6d4 | 15-20 | 7.66 | 0.39 | 0.57 | 50.21 | 148.51 | 0.03 | NA | NO |
| s6d5 | 20-25 | 7.71 | 0.50 | 0.45 | 53.87 | 159.78 | 0.03 | NA | NO |
| s6d6 | 25-30 | 7.57 | 0.55 | 0.47 | 56.49 | 121.38 | 0.03 | NA | NO |
| w1d1 | 0-5 | 7.47 | ND* | 0.86 | 56.49 | 158.77 | 1 | 20 | NO |
| w1d2 | 5-10 | 7.42 | ND | 0.61 | 50.46 | 102.01 | 1 | NA | NO |
| w1d3 | 10-15 | 7.47 | 0.12 | 0.79 | 54.53 | 101.33 | 1 | NA | NO |
| w1d4 | 15-20 | 7.50 | ND | 0.97 | 51.12 | 138.88 | 1 | NA | NO |
| w1d5 | 20-25 | 7.64 | ND | 0.40 | 55.78 | 104.13 | 1 | NA | NO |
| w1d6 | 25-30 | 7.60 | ND | 1.06 | 52.09 | 95.79 | 1 | NA | NO |
| w3d1 | 0-5 | 7.29 | ND | 1.53 | 50.43 | 98.39 | 0.7 | 20 | submerged green plants |
| w3d2 | 5-10 | 7.46 | ND | 0.86 | 44.55 | 117.91 | 0.7 | NA |  |
| w3d3 | 10-15 | 7.22 | ND | 1.30 | 36.12 | 112.37 | 0.7 | NA |  |
| w3d4 | 15-20 | 7.37 | ND | 3.33 | 35.55 | 119.42 | 0.7 | NA |  |
| w3d5 | 20-25 | 7.28 | ND | 4.36 | 30.27 | 114.29 | 0.7 | NA |  |
| w3d6 | 25-30 | 7.39 | ND | 4.70 | 35.08 | 105.87 | 0.7 | NA |  |
| w5d1 | 0-5 | 7.30 | ND | 1.33 | 51.55 | 115.52 | 0.5 | 20 | NO |
| w5d2 | 5-10 | 7.37 | 1.38 | 1.28 | 36.55 | 129.92 | 0.5 | NA | NO |
| w5d3 | 10-15 | 7.22 | ND | 1.33 | 36.64 | 123.86 | 0.5 | NA | NO |
| w5d4 | 15-20 | 7.30 | ND | 1.30 | 41.75 | 100.1 | 0.5 | NA | NO |
| w5d5 | 20-25 | 7.24 | ND | 0.96 | 36.54 | 126.27 | 0.5 | NA | NO |
| w5d6 | 25-30 | 7.31 | ND | 2.04 | 37.57 | 123.78 | 0.5 | NA | NO |
| w6d1 | 0-5 | 7.60 | ND | 57.40 | 52.04 | 190.11 | 0.03 | 20 | NO |
| w6d2 | 5-10 | 7.52 | ND | 74.50 | 48.40 | 211.17 | 0.03 | NA | NO |
| w6d3 | 10-15 | 7.46 | 0.75 | 64.90 | 47.98 | 176.87 | 0.03 | NA | NO |
| w6d4 | 15-20 | 7.43 | ND | 50.80 | 51.26 | 213.83 | 0.03 | NA | NO |
| w6d5 | 20-25 | 7.36 | 0.80 | 12.90 | 57.04 | 122.33 | 0.03 | NA | NO |
| w6d6 | 25-30 | 7.55 | ND | 1.85 | 60.32 | 107.19 | 0.03 | NA | NO |

ND, below 0.05 mg/L; NA, not analyzed; NO, not observed; SA, the same as above.

**Table S3 Alpha diversity indices of samples collected from the Huixian karst wetland in the summer and winter.**

| Sample | Observed OTUs | Chao1 | Ace | PD whole tree | Good's coverage | Simpson's index (D) | Shannon diversity index |
| --- | --- | --- | --- | --- | --- | --- | --- |
| s1d1 | 306 | 843.568181818 | 1025.12939991 | 17.38059 | 0.70013755 | 0.976900037652 | 7.06122607803 |
| s1d2 | 225 | 511.538461538 | 612.779165254 | 12.93661 | 0.70873786 | 0.977637854652 | 6.8687634858 |
| s1d3 | 210 | 400.65 | 442.074973718 | 13.02619 | 0.7936772 | 0.974631853179 | 6.61309359347 |
| s1d4 | 254 | 647.825 | 877.822395408 | 14.89197 | 0.67811935 | 0.985752544889 | 7.15875194989 |
| s1d5 | 190 | 578.166666667 | 645.725862206 | 12.22404 | 0.63466667 | 0.985841777778 | 6.92472838595 |
| s1d6 | 231 | 423.446808511 | 463.602377786 | 13.1179 | 0.75454545 | 0.984165289256 | 7.06395081904 |
| s3d1 | 177 | 510.375 | 582.135831515 | 10.8092 | 0.64124294 | 0.98206134891 | 6.75201484816 |
| s3d2 | 545 | 1078.93877551 | 1221.8748828 | 28.81524 | 0.79402416 | 0.991453440509 | 8.0409231534 |
| s3d3 | 210 | 506.129032258 | 566.282309939 | 13.10923 | 0.728 | 0.986232 | 6.96956960311 |
| s3d4 | 244 | 656.59375 | 679.9857181 | 15.723 | 0.72089041 | 0.985914336649 | 7.10633986195 |
| s3d5 | 156 | 513.0 | 545.123287671 | 11.04745 | 0.48275862 | 0.988592449465 | 6.96373521072 |
| s3d6 | 234 | 489.5 | 546.405366725 | 14.4386 | 0.72928177 | 0.987915848451 | 7.1481508681 |
| s5d1 | 131 | 392.625 | 396.782653338 | 8.17872 | 0.6838488 | 0.951169683872 | 5.98111157912 |
| s5d2 | 252 | 648.028571429 | 675.680144703 | 15.57115 | 0.70284698 | 0.988848925419 | 7.29474345522 |
| s5d3 | 263 | 653.384615385 | 724.30212051 | 15.20065 | 0.70034247 | 0.984659410771 | 7.2382785262 |
| s5d4 | 226 | 623.833333333 | 693.148555647 | 13.36854 | 0.67230444 | 0.989116296949 | 7.19502006413 |
| s5d5 | 243 | 573.972972973 | 612.656953812 | 12.95165 | 0.69573643 | 0.989250946458 | 7.32098990187 |
| s5d6 | 246 | 771.1 | 806.842495928 | 14.70687 | 0.63374486 | 0.98408101746 | 7.19343654856 |
| s6d1 | 270 | 742.777777778 | 818.995148356 | 15.04469 | 0.75974026 | 0.956950581886 | 6.5883924413 |
| s6d2 | 230 | 501.95 | 604.918478175 | 13.23068 | 0.73712256 | 0.981629118305 | 6.90383368137 |
| s6d3 | 228 | 690.033333333 | 826.164615559 | 14.02728 | 0.68667917 | 0.972110852585 | 6.62637578123 |
| s6d4 | 282 | 798.486486486 | 877.486463097 | 17.63046 | 0.70257967 | 0.984864638333 | 7.22149400388 |
| s6d5 | 232 | 618.571428571 | 773.91998293 | 13.74909 | 0.65189873 | 0.985427905072 | 7.1029347519 |
| s6d6 | 215 | 530.617647059 | 617.030214097 | 13.44248 | 0.73321234 | 0.975497445661 | 6.61476355579 |
| w1d1 | 338 | 918.021276596 | 961.999610125 | 22.32691 | 0.71146732 | 0.984481261812 | 7.32115479662 |
| w1d2 | 255 | 674.236842105 | 828.168339879 | 17.49369 | 0.70559211 | 0.980198234072 | 6.92485213377 |
| w1d3 | 234 | 729.933333333 | 914.644074382 | 16.15184 | 0.7092437 | 0.964779323494 | 6.47806478466 |
| w1d4 | 255 | 682.0 | 919.666178013 | 18.66134 | 0.7140625 | 0.970126953125 | 6.65235185339 |
| w1d5 | 246 | 970.625 | 1028.54021556 | 15.73787 | 0.6511194 | 0.978175818668 | 6.88533885131 |
| w1d6 | 486 | 1108.2 | 1216.6133348 | 28.23298 | 0.80359435 | 0.981584138184 | 7.4383971663 |
| w3d1 | 216 | 528.0 | 575.075325397 | 15.17244 | 0.68 | 0.988197530864 | 7.14065680504 |
| w3d2 | 724 | 1488.29411765 | 1589.75493764 | 40.81817 | 0.80852018 | 0.992221440206 | 8.32525297368 |
| w3d3 | 296 | 691.813953488 | 738.84081035 | 22.06182 | 0.82296651 | 0.976543577299 | 6.74353355332 |
| w3d4 | 390 | 831.230769231 | 877.28107254 | 22.75283 | 0.76630964 | 0.990019237169 | 7.67560080861 |
| w3d5 | 338 | 847.47826087 | 848.074120009 | 21.85954 | 0.73406863 | 0.988994617455 | 7.59455261769 |
| w3d6 | 366 | 832.381818182 | 885.477546801 | 23.34429 | 0.74889381 | 0.99186751116 | 7.75767242702 |
| w5d1 | 228 | 441.0 | 518.098711374 | 13.52471 | 0.69593148 | 0.988055335207 | 7.23774989369 |
| w5d2 | 279 | 673.8 | 770.371367252 | 17.00997 | 0.67130435 | 0.987493383743 | 7.38697080778 |
| w5d3 | 451 | 991.467532468 | 1160.602571 | 26.30492 | 0.77666151 | 0.97906996744 | 7.450875779 |
| w5d4 | 176 | 563.434782609 | 626.928630823 | 12.50021 | 0.53633218 | 0.986003520073 | 6.9648260072 |
| w5d5 | 276 | 917.935483871 | 936.509809106 | 17.6216 | 0.64349376 | 0.988030668433 | 7.38915776808 |
| w5d6 | 285 | 876.176470588 | 888.444146188 | 17.89328 | 0.66218487 | 0.987455688158 | 7.40665336762 |
| w6d1 | 282 | 644.23255814 | 658.114324096 | 16.68782 | 0.71904762 | 0.991730914588 | 7.54396612795 |
| w6d2 | 359 | 805.6 | 864.065754508 | 21.35366 | 0.68478261 | 0.993202829631 | 7.91195530853 |
| w6d3 | 381 | 879.620689655 | 980.549254414 | 22.01094 | 0.77476636 | 0.987693248319 | 7.50584046375 |
| w6d4 | 341 | 954.108695652 | 1017.88683079 | 21.62535 | 0.6744186 | 0.990016112703 | 7.62948267207 |
| w6d5 | 231 | 545.078947368 | 678.821258122 | 14.86407 | 0.77503628 | 0.950743699984 | 6.23392462709 |
| w6d6 | 410 | 1095.0 | 1184.0072052 | 23.47013 | 0.77678571 | 0.98343945016 | 7.29332329376 |

# 2 Supplementary Figure

#

**Supplementary Figure S1.** Phylogenetic tree of 16S rRNA gene OTU representative sequences which were assigned to anaerobic methanotrophic archaea ANME-2d recovered from Huixian karst wetland. The phylogeny was reconstructed using approximately-maximum-likelihood algorithm in FastTree 2.1.11. Branches of a total 140 representative sequences of ANME-2d OTUs observed in this study were highlighted in red, following by OTU number and sequences number, or sequences number only. The stability of the tree is evaluated by using the local support value (%), and local support values are shown only for important nodes. The scale bars represent 6% sequence divergence.
